# Human Papillomavirus-associated oropharyngeal cancer: an observational study of diagnosis, prevalence and prognosis in a UK population

**DOI:** 10.1186/1471-2407-13-220

**Published:** 2013-05-01

**Authors:** Mererid Evans, Robert Newcombe, Alison Fiander, James Powell, Martin Rolles, Selvam Thavaraj, Max Robinson, Ned Powell

**Affiliations:** 1 Velindre Cancer Centre, Whitchurch, Cardiff, CF14 2TL, UK; 2 Department of Primary Care and Public Health, School of Medicine, Cardiff University, Heath Park, Cardiff, CF14 4XN, UK; 3 Department of Obstetrics and Gynaecology, School of Medicine, Cardiff University, Heath Park, Cardiff, CF14 4XN, UK; 4 Singleton Hospital, Sketty Lane, Swansea,, SA2 8QA, UK; 5 Department of Clinical and Diagnostic Sciences, King’s College London Dental Institute, Strand, London, WC2R 2LS, UK; 6 Centre for Oral Health Research, Newcastle University, Framlington Place, Newcastle Upon Tyne, NE2 4BW, UK; 7 HPV Oncology Group, Institute of Cancer and Genetics, School of Medicine, Cardiff University, Heath Park, Cardiff, CF14 4XN, UK

**Keywords:** Oropharyngeal, Oropharynx, Cancer, HPV, Papillomavirus, Prognosis, Tonsil

## Abstract

**Background:**

The incidence of Human Papillomavirus (HPV) associated oropharyngeal cancer (OPC) is increasing. HPV-associated OPC appear to have better prognosis than HPV-negative OPC. The aim of this study was to robustly determine the prevalence of HPV-positive OPC in an unselected UK population and correlate HPV positivity with clinical outcome.

**Methods:**

HPV testing by GP5+/6+ PCR, In Situ Hybridisation (ISH) and p16 immunohistochemistry (IHC) was performed on 138 OPCs diagnosed in South Wales (UK) between 2001–06. Kaplan-Meier analysis was used to correlate HPV status with clinical outcome.

**Results:**

Using a composite definition of HPV positivity (HPV DNA and p16 overexpression), HPV was detected in 46/83 (55%) samples where DNA quality was assured. Five year overall survival was 75.4% (95% CI: 65.2 to 85.5) in HPV-positives vs 25.3% (95% CI: 14.2 to 36.4) in HPV negatives, corresponding to a 78% reduction in death rate (HR 0.22, p < 0.001). HPV-positives had less locoregional recurrence but second HPV-positive Head and Neck primaries occurred. Poor quality DNA in fixed pathological specimens reduced both HPV prevalence estimates and the prognostic utility of DNA-based HPV testing methods. As a single marker, p16 was least affected by sample quality and correlated well with prognosis, although was not sufficient on its own for accurate HPV prevalence reporting.

**Conclusions:**

This study highlights the significant burden of OPC associated with HPV infection. HPV positive cases are clinically distinct from other OPC, and are associated with significantly better clinical outcomes. A composite definition of HPV positivity should be used for accurate prevalence reporting and up-front DNA quality assessment is recommended for any DNA-based HPV detection strategy.

## Background

Squamous cell carcinoma of the oropharynx, affecting the tonsils, base of tongue, pharyngeal wall and soft palate, has increased in incidence in developed countries over the last 20 years [1,2]. This increase has been attributed to Human Papillomavirus (HPV). In Sweden, a doubling in tonsil cancer incidence prompted reports of an ‘epidemic of a virus-induced carcinoma’ [1]. HPV prevalence rates in oropharyngeal cancer (OPC) range from 36% to >80%, varying with geographical location and anatomical subsite [3-5]. It is likely that population-specific incidence rates of HPV-induced OPC are influenced by oral HPV infection rates, sexual behaviour, and rates of smoking and drinking. Among HPV positive OPC, HPV16 is the predominant genotype, accounting for approximately 95% of cases [6].

Whereas virtually all cervical cancers are HPV-induced, OPC has two distinct aetiologies: consumption of tobacco and alcohol, or HPV infection, which may co-exist [5]. Until recently, the relevant aetiological agent in an individual patient was unknown but making this distinction is clinically important because HPV-positive OPC is associated with better response to chemotherapy, chemoradiotherapy (CRT) and radiotherapy (RT) and has a better prognosis compared to HPV-negative OPC [6-10]. Better outcomes have also been reported after surgery, suggesting that improved outcomes in HPV-positive patients are independent of treatment received [3,11]. Other factors, particularly tobacco smoking, may adversely affect prognosis in HPV-positive OPC [5,7].

A variety of HPV detection methods are available and differences in test characteristics may partly explain the variation in HPV prevalence rates reported in different studies [12]. Because the presence of HPV DNA in a tumour *per se* is not evidence of a causal relationship, a marker of HPV activity is needed to diagnose HPV-induced OPC. A widely used ‘surrogate’ biomarker of HPV activity, is p16 immunohistochemistry (IHC). p16 is a cyclin-dependent-kinase inhibitor and is induced as a consequence of inhibition of Rb activity by the HPV E7 oncoprotein (in most other Head and Neck (H&N) cancers, p16 is down regulated). Two main diagnostic algorithms have emerged for use in the clinical setting: both advocate screening by p16 IHC followed by detection of HPV DNA, either by consensus PCR or In Situ Hybridization (ISH) [13,14].

This study aimed to determine the prevalence of HPV-associated OPC in South Wales (UK) and investigate the diagnostic and prognostic utility of GP5+/6+ PCR enzyme immunoassay, ISH and p16 IHC. Most published data relating HPV status with clinical outcome is based on clinical trial cohorts and, while this enables collection of high quality clinical data, it results in exclusion of some patient groups, including palliative patients. This study did not systematically exclude any patients and demonstrates the impact of HPV status on outcome in a ‘real-world’ population of patients with OPC. This provides insight into the behaviour and late outcomes of HPV-positive OPC.

## Methods

### Study population

Patients diagnosed with OPC (ICD-10 codes C01, C05.1, C05.2, C09, C10) in South Wales (UK) 1/9/2001-31/8/2006 were identified from pathology databases. Data on clinicopathological characteristics and outcome were obtained from an electronic health record used at the regional Cancer Centre. Deaths in peripheral hospitals were automatically fed into the electronic record. Where cause of death was not documented on the electronic record, it was elucidated by review of patient notes, review of clinic letters and/or discussion with General Practitioners. For every patient who was alive at the point of analysis but had not been seen in hospital for the preceding 12 months (eg had been discharged from follow-up), the study team contacted the General Practitioner to ensure that the patient was indeed still alive with no evidence of disease recurrence. Where smoking history was available, patients were classified as current, never or previous smokers (stopped smoking >3 months before diagnosis). Locoregional recurrence was defined as recurrence at the primary site and/or cervical lymph nodes after a complete response to treatment.

One representative formalin fixed paraffin embedded (FFPE) block was retrieved for each case. Histological diagnosis of squamous carcinoma of the oropharynx was confirmed by two pathologists with special interest in OPC (MR and ST).

Approval for the study was obtained from South East Wales Research Ethics Committee (ref: 09/WSE03/44).

### HPV detection

#### DNA extraction and assessment of sample adequacy

Sectioning was performed with appropriate precautions to prevent inter-block DNA contamination (eg thorough cleaning of microtome, use of fresh blades). DNA was extracted from 2 × 10 μm sections of FFPE biopsies using the Qiagen FFPE Kit (Qiagen, Hilden, Germany). DNA quality was assessed by PCR for a 119 bp fragment of the human HMBS gene. To control for contamination during sectioning, regular sections were cut from a blank paraffin block and processed in parallel with the tumour sections. Positive (HPV16 positive Caski cell line DNA) and negative (water) controls were included for each PCR run. All blanks and negative controls tested negative for HMBS and HPV DNA.

### GP5+/6+ PCR enzyme immune assay (EIA)

Samples were genotyped for HPV DNA by GP5+/6+ PCR EIA. HPV typing was performed in 2 stages the first stage used cocktails of probes for 14 high risk and 6 low risk HPV types; PCR was then repeated on positive samples, which were then typed with individual probes [15]. This assay detects DNA from high risk HPV types: 16, 18, 31, 33, 35, 39, 45, 51, 52, 56, 58, 59, 66 and 68; and low risk types: 6, 11, 40, 42, 43 and 44. Full details of the HMBS PCR and GP5+/6+ PCR EIA are provided in Additional file 1.

### High-risk HPV in situ hybridisation

High-risk HPV ISH was performed using the Inform HPV III Family 16 Probe, (Ventana Medical Systems Inc, USA) on a Benchmark Autostainer (Ventana Medical Systems) for HPV types 16, 18, 31, 33, 35, 39, 51, 52, 56, 58 and 66. CaSki cells (HPV16 positive; 200–400 copies/cell), HeLa cells (HPV18 positive; 10–50 copies/cell) and C-33A (HPV negative) were used as controls. The HR-HPV ISH test was scored as positive if blue reaction product colocalised with the nuclei of malignant cells. Diffuse nuclear and cytoplasmic staining and punctate nuclear staining were scored as positive. Focal specific staining of only part of the tumour section was regarded as positive. Diffuse staining of tumour and stromal tissues, considered to represent non-specific chromogen precipitate, was scored as negative.

### p16 immunohistochemistry

p16 IHC was carried out using the CINtec Histology kit (mtm Laboratories, AG, Germany) on a Ventana Benchmark Autostainer. A tonsil SCC with high p16 expression was used as a positive control. The primary antibody was omitted from negative controls. p16 IHC was scored as positive if there was strong and diffuse nuclear and cytoplasmic staining present in greater than 70% of the malignant cells. All other staining patterns were scored as negative. All samples were scored independently by two expert H&N pathologists and discordant cases were reviewed to come to a consensus score. Both ISH and p16 IHC were carried out at the Department of Cellular Pathology, Newcastle, UK as previously described [14].

### Interpretation of HPV test results

A binary classification (positive vs negative) was used to score the p16 IHC and HPV ISH. Stained sections were assessed independently by two pathologists, who met to resolve discordant interpretations and establish a consensus categorization. For PCR-EIA, positivity was defined as giving an absorbance at 405 nm of greater than three times background.

### Statistical methods

Overall Survival (OS) analyses were based on time from diagnosis to death; survivors were censored at their last follow-up. Progression Free Survival (PFS) analyses were based on time from diagnosis to first event (locoregional recurrence, distant metastasis or death from any cause); patients without an event were censored at their last follow-up. Analyses of OS and PFS included all patients, irrespective of treatment intent and response to treatment. Kaplan-Meier analysis was used to obtain survival plots and 3- and 5-year survival. The Cox proportional hazards model was used to estimate Hazard Ratios (HR) characterising the independent prognostic significance of single and multiple variables, namely HPV and smoking status/treatment method. Further analyses for variables including age and stage were not performed as sub-groups were insufficient to be statistically robust.

## Results

Histology blocks were obtained for 147 cases, representing 83% of patients diagnosed with OPC in South Wales during the period. Nine blocks did not contain sufficient tumour for analysis. Analyses are presented for 138 patients with histologically confirmed squamous OPC.

### HPV prevalence

Tumours were classified as HPV-positive if they contained HPV DNA (by GP5+/6+ PCR and/or ISH) and overexpressed p16 [13,14]. Four groups were defined: ‘true’ HPV positives, ‘true’ HPV negatives and two ‘equivocal’ groups (Table 1) as described by Weinberger *et al.*[16].

**Table 1:** Baseline characteristics of patients and tumours grouped by HPV results

	**All 138 patients**	**Group 1 (n = 59) ‘True’ negatives p16 negative PCR & ISH negative**	**Group 2 (n = 6) 'Equivocal' p16 negative PCR &/or ISH positive**	**Group 3 (n = 69) ‘True’ positives p16 positive PCR &/or ISH positive**	**Group 4 (n = 4) 'Equivocal' p16 positive PCR & ISH negative**	**Test comparing groups 1 & 3**
Male	104 (75%)	44 (75%)	5	51 (74%)	4	X^2^_1_: p = 0.93
Female	34 (25%)	15 (25%)	1	18 (26%)	0	
Age in years Mean (SD)	58.1 (10.7)	61.6 (10.1)	57 (7.9)	55.7 (10.9)	48.8 (0.5)	t: p = 0.002
Smoking*						
Current	63 (55%)	41 (80%)	4	18 (32%)	0	X^2^_2_: p < 0.001
Previous	32 (28%)	9 (18%)	0	23 (41%)	0	
Never	20 (17%)	1 (2%)	2	15 (27%)	2	
**Performance status**						
0	68 (49%)	15 (25%)	4	46 (67%)	3	MW: p < 0.001
1	42 (30%)	21 (36%)	1	19 (28%)	1	
2	21 (15%)	17 (29%)	0	4 (6%)	0	
3	7 (5%)	6 (10%)	1	0 (0%)	0	
**Primary tumour site**						
Tonsil	93 (67%)	33 (56%)	4	54 (78%)	2	X^2^_2_: p < 0.001
Tongue base or vallecula	35 (25%)	16 (27%)	2	15 (22%)	2	
Other^1^	10 (7%)	10 (17%)	0	0 (0%)	0	
**AJCC stage**						
I	5 (4%)	5 (8%)	0	0 (0%)	0	MW: p = 0.53
II	5 (4%)	3 (5%)	0	2 (3%)	0	
III	28 (20%)	8 (14%)	0	20 (29%)	0	
IVA	87 (63%)	35 (59%)	5	43 (62%)	4	
IVB	8 (6%)	3 (5%)	1	4 (6%)	0	
IVC	5 (4%)	5 (8%)	0	0 (0%)	0	
**Primary treatment**						
Surgery	71(51%)	21 (36%)	4	42 (61%)	4	X^2^_1_: p = 0.07
Radiotherapy	55 (40%)	27 (46%)	1	27 (39%)	0	
Radical	126 (91%)	48 (81%)	5	69 (100%)	4	X^2^_1_: p = 0.001
Palliative	12 (9%)	11 (19%)	1	0 (0%)	0	
**DNA Adequacy**						
HMBS positive	83	32 (39%)	4 (5%)	46 (55.4%)	1 (1%)	
HMBS negative	55	27 (49%)	2 (4%)	23 (42%)	3 (5%)	
All cases	138	59 (43%)	6 (4%)	69 (50%)	4 (3%)	

Tests used: X^2^_1_ - chi-square on 1 degree of freedom (df); X^2^_2_ - chi-square on 2 df; t- independent samples *t*-test; MW Mann–Whitney test.

AJCC - American Joint Committee on Cancer, SD - Standard deviation.

* Analyses for smoking exclude 23 cases with smoking history unknown.

^1^Other: includes soft palate, uvula and posterior pharyngeal wall.

The overall HPV prevalence rate was 55% (46/83) (95% CI: 45–66) when DNA quality was assured. HPV prevalence fell to 50% (69/138) (95% CI: 42–58) if HMBS negative cases were included, consistent with the occurrence of some HPV DNA false negative results in samples containing poor quality DNA (see below).

Among cases that tested positive for HPV by GP5+/6+ PCR, 97% (67/69) were positive for HPV16. One case contained HPV33 and one case showed co-infection with HPV18 and HPV56. No low-risk HPV infections were detected.

### Influence of DNA quality on HPV detection rate

DNA quality was assessed by PCR for the human HMBS gene which was amplifiable in 83/138 cases (60%), suggesting high levels of DNA degradation in the other samples. DNA adequacy ranged from 30% in hospitals using unbuffered formalin as a fixative to 96% in those using neutral buffered formalin.

HPV testing was carried out on all samples regardless of DNA quality. This revealed a high false negative rate when DNA-based HPV detection methods (PCR and ISH) were used on samples containing poor quality DNA (HMBS negative) e.g. HPV DNA positivity by GP5+/6+ PCR was 23% lower in HMBS negative than HMBS positive cases. DNA degradation did not have a significant effect on p16 IHC testing results. Estimated false negative rates are shown in Table 2.

**Table 2:** Estimation of false negatives among 55 HMBS negative cases

**HPV marker**	**Observed HPV positives among**		**Chi-square test for association between observed HPV status and HMBS**	**Estimated false negatives for HPV among 55 HMBS negative cases**		
	**HMBS positive n = 83**	**HMBS negative n = 55**		**Number**	**As proportion of 55 HMBS negative cases**	**95% confidence interval**
GP5+/6+ PCR	49 (59%)	20 (36%)	p = 0.009	12.5	23%	6% to 38%
ISH	42 (51%)	18 (33%)	p = 0.04	9.8	18%	1% to 33%
p16	47 (57%)	26 (47%)	p = 0.28	5.1	9%	0%* to 26%

For GP5+/6+ PCR, number of false negatives estimated as 49 × 55/83 – 20 = 12.5, representing 23% of HMBS negative cases. The same method was used to calculate false negative rates for ISH and p16.

*95% confidence interval extends below 0 for p16 – shown truncated here.

### Agreement between HPV testing methods

In samples with good quality DNA (HMBS positive), the proportion of positive samples was similar when analysed by p16 (47/83 cases, 57%) and GP5+/6+ PCR (49/83, 59%) and slightly lower using ISH (42/83, 51%). When samples containing poor quality DNA were included, prevalence rates by GP5+/6+ PCR and ISH were lower (50% and 43% respectively), consistent with the presence of false negatives in this group. Concordance between tests was highest for p16 and GP5+/6+ PCR (5% of cases discordant) than for either test with ISH (11% discordant for each). The number of discordant cases increased for each comparison if HMBS negatives were included showing that poor DNA quality reduced consistency between HPV testing results, as well as overall estimates of HPV prevalence. Analysis of concordance is shown in Table 3.

**Table 3:** Concordance between HPV test results

**P16**	**GP5+/6+**	**All cases (n = 138)**		**HMBS positive cases only (n = 83)**	
		**ISH -**	**ISH +**	**ISH -**	**ISH +**
-	-	59	1	32	1
-	+	3	2	2	1
+	-	4	5	1	0
+	+	12	52	6	40

The table shows results of the three tests for all cases and for HMBS positive cases only. (−) indicates a negative test result; (+) indicates a positive test result.

### Prognostic value of HPV testing methods

Individually, each HPV testing method was highly prognostic for Overall Survival (OS) and Progression Free Survival (PFS). When all cases were included, p16 correlated well with prognosis (point estimate of HR for death 0.24, 95% CI 0.15-0.39), as did ISH (0.27, 95% CI 0.16-0.46) and GP5+/6+ PCR (0.29, 95% CI 0.18-0.47), although all were slightly inferior to the composite definition of HPV-positivity (0.22, 95% CI 0.13-0.37), and no test performed significantly better than another. If HMBS negative cases were excluded, the prognostic value of GP5+/6+ PCR improved to equal that of the composite marker and p16 (HR 0.20), suggesting that GP5+/6+ PCR and p16 are equally prognostic when DNA quality is assured. Hazard Ratios are shown in Table 4.

**Table 4:** Prognostic ability of individual HPV detection methods

**Group**	**p16**	**ISH**	**GP5+/6+ PCR**
All cases (n = 138)	0.24 (0.15-0.39)	0.27 (0.16-0.46)	0.29 (0.18-0.47)
All cases excluding equivocals (n = 128)	0.22* (0.13-0.37)	0.25 (0.14-0.43)	0.26 (0.16-0.44)
HMBS positive cases (n = 83)	0.20 (0.10-0.37)	0.33 (0.18-0.62)	0.22 (0.12-0.41)
HMBS positive cases excluding equivocals (n = 78)	0.20* (0.10-0.38)	0.27 (0.14-0.53)	0.20 (0.10-0.38)

Hazard ratios (HR) for overall survival, with 95% confidence intervals, are shown for each HPV testing method.

* When ‘equivocal’ cases (with discrepant p16 and HPV DNA testing results) are excluded, analyses for p16 and the composite criterion for HPV positivity based on 3 markers are equivalent and represent the ‘gold standard’.

### Patient characteristics

Baseline patient and tumour characteristics are shown in Table 1. The proportion of HPV-positive cases was similar in men and women, although men were more frequently affected overall (75% cases). HPV-positive patients were 6 years younger (p = 0.002), had better performance status (p < 0.001) and were less likely to be current smokers (p < 0.001) than HPV-negative patients. HPV-positive cancers occurred exclusively in the tonsil (78%) and tongue base (22%), whereas 17% of HPV-negative cancers arose elsewhere in the oropharynx (soft palate, uvula, posterior pharyngeal wall). Overall disease stage was similar, although HPV-positive patients were less likely to present with distant metastases (0% vs 8%, p = 0.04). HPV-positive patients were treated radically (curatively) more often than HPV-negative patients (p < 0.001), 19% of whom were treated palliatively. Of radically treated patients, 54% underwent primary surgery (+/− post-operative RT) and 46% underwent primary RT (+/− chemotherapy, CRT), reflecting local practice at the time. There was a trend for more HPV-positive cases to have primary surgery (42/69 cases, 61%) and more HPV-negative cases to have primary RT/CRT (27/48, 56%) (p = 0.068). Patients treated with RT/CRT were older (mean age 61 years) and had poorer performance status than those treated surgically (mean age 52 years), in keeping with different HPV prevalence in both groups.

### Effect of HPV on survival and recurrence

After median follow-up from diagnosis of 4.9 years (range 0.1 to 10.1 years), 77 deaths occurred in 138 patients. Overall Survival (OS) by Kaplan-Meier survival analysis was 59.4% at 3 years (95% CI: 51.2 to 67.6) and 52.2% at 5 years (95% CI: 43.8 to 60.5). No significant difference in survival was seen when HMBS negative cases were excluded from the analysis and, as a result, HMBS positive and negative cases were combined for subsequent analyses, although every analysis was repeated in HMBS positive cases only to ensure that the results were consistent (data not shown). For 126 radically treated patients, OS was 65.1% at 3 years and 57.1% at 5 years. For the 12 palliative patients, median survival was 186 days (6 months), range 28–802 days.

**Figure 1:**
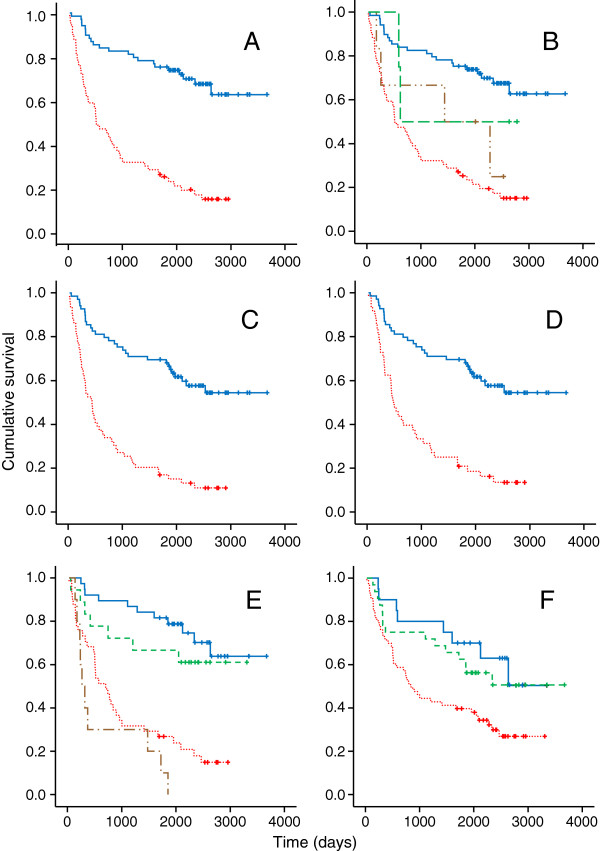
**Kaplan-Meier plots. A**. Overall survival by HPV status. Blue solid line: HPV-positive patients (n = 69), red dotted line: HPV-negative patients (n = 59). 10 patients with equivocal HPV status are excluded. **B**. Overall survival in 4 groups classified by p16 expression and presence of HPV DNA. Blue solid line: HPV-positive (Group 3, n = 69), brown dashed/dotted line: p16 negative and ISH/PCR positive (Group 2, n = 6), green dashed line: p16 positive and ISH/PCR negative (Group 4, n = 4), red dotted line: HPV negative (Group 1, n = 59). **C**. Progression free survival by HPV status. Blue solid line: HPV-positive patients (n = 69), red dotted line: HPV-negative patients (n = 59). 10 patients with equivocal HPV status are excluded. **D**. Progression free survival in radically treated patients by HPV status. Blue solid line: HPV-positive patients (n = 69), red dotted line: HPV-negative patients (n = 48). 21 patients with equivocal HPV status or palliative intent are excluded. **E**. Overall survival by HPV and smoking status. Blue solid line: HPV-positive not current smokers (n = 38), green dashed line: HPV-positive current smokers (n = 18), red dotted line: HPV-negative current smokers (n = 41), brown dotted/dashed line: HPV-negative not current smokers (n = 10). Cases with equivocal HPV status or unknown smoking status are excluded. **F**. Overall survival by smoking status. Blue solid line: non-smokers (n = 20), green dashed line: previous smokers (n = 32), red dotted line: current smokers (n = 63). Cases with unknown smoking status are excluded.

A clear association between HPV-positivity and favourable prognosis was demonstrated in Kaplan-Meier analysis (Figure 1A). 3 and 5-year OS rates were 82.6% (95% CI: 73.7 to 91.5) and 75.4% (95% CI: 65.2 to 85.5) respectively in HPV-positive patients, compared to 32.2% (95% CI: 20.3 to 44.1) and 25.3% (95% CI: 14.2 to 36.4) in HPV-negative patients, corresponding to a 78% reduction in death rate associated with HPV-positivity (HR 0.220, 95% CI; 0.132-0.366, p < 0.001). Survival in patients with equivocal HPV status (n = 10) was intermediate between that of ‘true’ HPV-positives and negatives (Figure 1B). The effect of HPV status remained highly significant when palliative patients were excluded; OS at 3 and 5-years in radically treated HPV-positive patients was 82.6% and 75.4%, compared to 39.6% (95% CI: 32.5-46.7) and 31.1% (95% CI: 24.4-37.6) in HPV-negative patients, corresponding to a 74% reduction in the death rate (HR 0.259, 95% CI 0.152-0.440, p < 0.001).

At last follow-up, 87 patients (63%) had suffered an event (progression, recurrence or death). For HPV-positive patients, 3 and 5-year Progression Free Survival (PFS) rates were 72.5% (95% CI: 61.9 to 83.0) and 68.1% (95% CI: 57.1 to 69.1), compared to 25.4% (95% CI: 14.3 to 36.5) and 17% (95% CI 7.4 to 26.5) in HPV negatives, corresponding to a 75% reduction in the rate of progression, relapse or death associated with HPV-positivity (HR 0.25, 95% CI 0.15 to 0.39, p < 0.001) (Figure 1C). As with OS, PFS in patients with equivocal HPV status was intermediate between that of ‘true’ HPV-positives and negatives. PFS at 3 and 5-years in radically treated HPV-positive patients was 72.5% and 68.1%, compared to 31.2% and 20.8% in HPV-negative patients, corresponding to a 72% reduction in the rate of relapse or death (HR 0.28, 95% CI 0.17-0.46, p < 0.001) (Figure 1D).

Kaplan-Meier analysis of survival by HPV and smoking status simultaneously demonstrated a significant survival advantage associated with HPV-positivity, regardless of smoking status (Figure 1E). Survival was significantly better in previous and never smokers than in current smokers (HR for death by not current smoking 0.48, 95% CI 0.28-0.81, p = 0.006) (Figure 1F); Cox regression analysis showed that this was due entirely to their tendency to be HPV-positive. HPV-positivity was also associated with better survival regardless of primary treatment modality (surgery or RT/CRT). Although OS and PFS were better in surgically treated patients overall than those treated with primary RT/CRT (HR 0.5 for surgery; 95% CI: 0.3-0.83, p = 0.007), a higher proportion of the surgical group were HPV-positive (66.7% vs 50%, p = 0.068); using Cox regression analysis to adjust for HPV status, the survival difference between the two groups was no longer statistically significant (HR 0.74, p = 0.36).

Locoregional recurrence rates were lower in HPV-positive than HPV-negative patients (8/69, 12% vs 16/59, 27%) whereas distant metastases occurred at similar rates (4/69, 8% vs 5/59, 6%), mostly in lungs/bones (Table 5). Second primary cancers occurred in 12% of patients overall, only slightly more frequently in HPV-negative (8/59, 13.6%) than HPV-positive (8/69, 11.6%) patients. Almost all second primaries in HPV-negative patients (7/8) occurred outside the H&N (mostly in the lung); the single case arising in the H&N (oropharynx) was HPV-negative. In contrast, three (3/8) second primaries occurred in the H&N in HPV-positive patients (1 sarcoma in a previous RT field was not analysed further): 1 patient developed a HPV16-positive poorly differentiated squamous carcinoma of the nasopharynx (negative for EBV on EBER ISH) 7 months after a HPV16-positive tonsillar cancer and 1 patient developed a HPV16-positive squamous carcinoma of the tongue base 7 years after a HPV16-positive tonsillar cancer.

**Table 5:** Second primary malignancy and recurrence (local/distant) by HPV status

**HPV status**	**All cases**	**Site of recurrence**				**Second Primary Malignancy (SPM)**		
		**None**	**H&N**	**DM**	**HN + DM**	**No SPM**	**SPM H&N**	**Other SPM**
**‘True’ negatives (Group 1)** p16 negative, PCR & ISH negative	59	39	15	4	1	51	1	7
**‘Equivocal' (Group 2)** p16 negative, PCR &/or ISH positive	6	4	1	1	0	5	0	1
**‘True’ positives (Group 3)** p16 positive, PCR &/or ISH positive	69	57	8	4	0	61	3	5
**‘Equivocal' (Group 4)** p16 positive, PCR & ISH negative	4	2	1	1	0	4	0	0
Total	138	102	25	10	1	121	4	13

Head and Neck (H&N), Distant Metastases (DM), Second Primary Malignancy (SPM).

## Discussion

Biologically relevant HPV infection, defined as presence of HPV DNA by ISH and/or PCR and p16 over-expression, was identified in 55% of patients diagnosed with OPC in South Wales (UK), 2001–2006. The survival advantage afforded by HPV in a ‘real world’ population of patients with OPC, including those managed palliatively, is clearly demonstrated as is the effect of HPV on the long-term clinical behaviour of the disease. The effect of poor quality DNA in fixed pathological specimens on the diagnostic and prognostic utility of DNA-based HPV testing methods, including ISH, is shown for the first time. P16 expression is not affected by DNA quality and may be utilized as a single marker of HPV infection in clinical practice, although a composite definition of HPV positivity is recommended for accurate HPV prevalence reporting.

HPV prevalence rates differ between different geographical regions and time periods. The rate in this study (55%) is consistent with international (51.2% (88/172)) and US series (59.4% (192/323)) collected between 2002–2005 [7,17]. It also adds to a picture of regional and temporal variation in HPV prevalence across the UK where rates of 37.5% (33/88) and 42.7% (77/180) have been reported [12,18]. The number of ‘equivocal’ cases with discrepant HPV DNA and p16 testing results was significantly lower than in some other studies [7,18], suggesting that a testing algorithm combining PCR and ISH increases sensitivity for HPV DNA detection [14]. Discordant HPV DNA and p16 testing results occurred in 6-7% of cases showing that p16 alone is not sufficient for studies that aim to accurately report HPV prevalence.

Poor quality DNA significantly reduced HPV prevalence estimates using PCR and ISH-based techniques, because of the occurrence of false negative results in samples containing degraded DNA. Although PCR-based testing protocols routinely incorporate assessment of DNA quality, DNA-based ISH techniques do not, and therefore risk under-estimating HPV prevalence; this may partly explain the lower sensitivity reported for ISH compared to other HPV detection methods in previous studies [17]. However a recently developed RNA-based ISH test for HPV does include a control for sample quality and shows considerable promise as a diagnostic marker for OPC [19]. The three HPV testing methods evaluated in this study were all good markers of survival, with no test performing significantly better than another. Hazard ratios (HR) for death were: 0.24 for p16, 0.27 for ISH, 0.29 for GP5+/6+ PCR and 0.22 for the composite definition of HPV positivity. Poor quality DNA reduced the prognostic value of DNA-based HPV testing methods and when poor quality samples were excluded, the prognostic value of GP5+/6+ PCR was similar to that of p16 and the composite marker (0.22). The effect of poor quality DNA on prevalence and prognostication is reduced by using the composite definition of HPV-positivity.

The clinical implications of HPV positivity in this unselected population of patients are clear. HPV positivity was associated with a 78% reduction in death rate (HR 0.22) and a 75% reduction in rate of progression, relapse or death (HR 0.25). This effect is greater than in many clinical trial cohorts, due in part (but not entirely) to the inclusion of palliative patients. Survival of radically treated HPV-positive patients was comparable to that reported in a large US study; 3y OS was 82.6% (95% CI: 73.7 to 91.5) compared to 82.4% (95% CI: 77.2-87.6) and 3y PFS was 72.5% (95% CI: 61.9 to 83.0) compared to 73.7% (95% CI: 67.7 to 79.8). Survival of radically treated HPV-negative patients was however significantly worse; 3y OS was 39.6% (95% CI: 32.5 to 46.7) compared to 57.1% (95% CI: 48.1-66.1) and 3y PFS was 31.2% (95% CI: 18.3 to 44.1) compared to 43.4% (95% CI: 34.4-52.4) [7]. Similarly low survival figures for HPV-negative OPC have been reported previously [20] and poor performance status (~30% would have been excluded from the US study on this basis) and infrequent use of concurrent chemotherapy (<30% vs 100% in the US study) may have affected outcome in this study. Their prognosis was poor regardless of whether they were treated with primary surgery or RT/CRT.

The excellent outcomes of HPV-positive patients were independent of smoking status or treatment method. Retrospective analyses have suggested that smoking can negatively affect survival in some HPV-positive patients [5,7,21], and this data has influenced the design of several clinical trials. Although the relatively small cohort (n = 117) with known smoking history, crude definition of smoking and/or large effect of HPV status on outcome may have masked the effect of smoking in this study, it is possible that the effect of smoking, particularly past smoking, on outcome from HPV-positive OPC has previously been over-estimated, and this issue must be addressed prospectively in future studies. There was a trend for more HPV-positive patients to undergo primary surgery in this study (p = 0.07); although HPV status was unknown when treatment decisions were made, it is likely that selection of younger, fitter patients for surgery, resulted in preferential selection of HPV-positive patients. This highlights the dangers of comparing outcomes from non-randomized studies of surgery and RT/CRT, without knowledge of HPV status; randomized trials with mandatory HPV testing are required to assess treatments for OPC in future.

Improved outcomes in HPV-positive patients reflected better locoregional control rates. In contrast, rates of distant metastases occurring on follow-up were similar in both HPV-positive and negative patients. The occurrence of second HPV16-positive primaries, both in the tongue base and nasopharynx (EBV-negative) in this study is intriguing. Second HPV-associated cancers in the tonsils and nasopharynx and HPV-positive/EBV-negative nasopharyngeal carcinomas have previously been reported [22-24] and it is possible that the lymphoid tissue throughout Waldeyer’s ring is particularly susceptible to HPV-induced transformation. Second primaries occurring in patients with a history of HPV-positive OPC should be tested for HPV and further studies to investigate the frequency and timing of HPV-positive second H&N primaries are required to inform future follow-up protocols.

There are several potential limitations to the study. Histology blocks for 83% of OPC patients presenting across South Wales over the study period were included. There were several reasons why other cases were not included: blocks were not collected from a number of smaller centres, there was limited collection from one major centre due to logistical difficulty in identifying the relevant cases, mismatches were observed in coding between registry and pathology databases, and some blocks were missing from pathology archives. There is no reason to suspect systematic bias in the sample, especially given the multi-factorial reasons for samples not being included, but the potential for some bias cannot be completely excluded. The proportion of HPV-positive OPC is likely to have increased since 2006 and the sample has limited geographical representation, thus whilst it adds to the picture of HPV prevalence in the UK, caution should be exercised in generalising the findings.

## Conclusions

HPV was responsible for the development of 55% of OPCs in this study. Significantly better locoregional control and survival were seen in HPV-positive cases. Given the substantial difference in prognosis, routine assessment of HPV status should be mandated in clinical practice. Standardisation of tests is clearly a significant issue but, as a single marker, p16 IHC appears prognostic and is unaffected by sample DNA quality, making it a useful test in clinical practice. P16 as a single marker is not sufficient however for studies which aim to accurately report HPV prevalence, when p16 coupled to at least one test for HPV DNA (PCR/ISH) is recommended. Based on the study results, it is recommended that any DNA-based HPV detection strategy should incorporate up-front DNA quality assessment to reduce the effect of poor DNA quality on HPV prevalence estimates.

## Abbreviations

HPV: Human Papillomavirus; OPC: Oropharyngeal Cancer; ISH: In Situ Hybridisation; IHC: Immunohisto chemistry; CRT: Chemoradiotherapy; RT: Radiotherapy; FFPE: Formalin Fixed Paraffin Embedded; EIA: Enzyme Immunoassay; HR: Hazard Ratio; OS: Overall Survival; PFS: Progression Free Survival.

## Competing interests

ME, AF and NP have received research funding, honoraria and support to attend conferences from companies that manufacture prophylactic HPV vaccines. The other authors declare that they have no competing interests.

## Authors’ contributions

ME conceived the study, obtained funding and drafted the manuscript. RN performed the statistical analyses. AF assisted in design of the study and drafting of the manuscript. JP identified patients and collated clinical data. MR identified patients and collated clinical data. ST and MR performed the histopathological analyses. NP assisted in design of the study, performed the PCR analyses and helped draft the manuscript. All authors read and approved the final manuscript.

## Pre-publication history

The pre-publication history for this paper can be accessed here:

http://www.biomedcentral.com/1471-2407/13/220/prepub

## Supplementary Material

Additional file 1 GP 5+/6+ PCR-EIA and HMBS PCR. Click here for file
