# Supplementary material for: Human Papillomavirus-associated oropharyngeal cancer: an observational study of diagnosis, prevalence and prognosis in a UK population
Source: BMC Cancer. 2013 May 1;13:220. doi: 10.1186/1471-2407-13-220 (PMC3644265; doi:10.1186/1471-2407-13-220)
Supplement: Additional file 1 — GP 5+/6+ PCR-EIA and HMBS PCR. [file 1471-2407-13-220-S1.docx]

**Additional file 1.**

**GP 5+/6+ PCR-EIA and HMBS PCR**

The GP5/6 PCR-EIA assay was performed as initially described in:

A general primer GP5+/GP6(+)-mediated PCR-enzyme immunoassay method for rapid detection of 14 high-risk and 6 low-risk human papillomavirus genotypes in cervical scrapings. Jacobs, M.V., Snijders, P.J., van den Brule, A.J., Helmerhorst, T.J., Meijer, C.J. & Walboomers, J.M. (1997). J Clin Microbiol, 35, 791-5.

A two tier method was developed and applied to each sample. This was composed of:

1. An initial ‘HR LR’ PCR-ELISA with a cocktail of type-specific probes divided into ‘HR’ and ‘LR’ HPV infection.

2. Sub-typing of all ‘HR’ positive samples with the following individual 14 ‘HR’ HPV probes: HPV 16; 18; 31; 33; 35; 39; 45; 51; 52; 56; 58; 59; 66; and 68.

#### GP5+/6+ PCR

The initial ‘HR LR’ and sub-typing PCR reaction were performed in final volumes of 25µl and 100µl respectively.

The primer sequences (5’-3’) were:

GP5+ - TTTGTTACTGTGGTAGATACTAC;

GP6+ - Biotin-GAAAAATAAACTGTAAATCATATTC

GP 5+/6+ 1 x 25 µl reaction (µl)

dNTPs 2mM 2.5

10 x PCR buffer 2.5

MgCl2 (50mM) 1.75

GP5+ primer (5μM) 2.5

GP6+bio primer (5μM) 2.5

H2O 8.15

Taq (Invitrogen) 0.1

DNA 5

PCR cycling conditions were 94°c - 4mins, then 40 cycles of 94°c - 30s, 40°c - 90s, 72°c - 60s followed by 72°c – 4mins.

#### HPV EIA

The HPV Enzyme Immuno Assay (EIA) involves DNA capture and denaturation, probe hybridisation and detection of bound probe.

DNA capture: 5μl of the HPV amplified PCR product was added to the appropriate well in a 96-well streptawell plate (Roche). 50μl 1xSSC/0.5%Tween was added to each well (800ml dH2O + 200ml 5X wash buffer) the plate was sealed and incubated at 37°C for 1hr.

Denaturation: Plates were then washed 3 times with 1xSSC / 0.5%Tween and excess liquid tapped from plate. 100μl 0.2N NaOH was added to each well and then the plate re-sealed and incubated at room temp for 15min.

Probe Hybridisation: Plates were washed a further 3 times with 1xSSC / 0.5%Tween and excess liquid removed. 50μl of the appropriate probe mixture was added and the plate was re-sealed and incubated at 37°c for 1hr.

Detection: Plates were washed another 3 times with 1xSSC / 0.5%Tween and excess liquid removed. 50μl diluted (1:5000) anti-dig Alkaline Phosphatase conjugate was added, plates re-sealed and incubated at 37°c for 1hr. Plates were then washed 5 times with 1xSSC / 0.5%Tween and 100μl substrate solution (pNPP solution - Sigma) added. Plates were sealed and incubated at 37°C for 1hr. Dual optical density readings were then taken at 415nm and 630nm at 1, 2 and 24hrs.

Results were analysed in a preformatted excel worksheet. Raw data from each reading at each time point was pasted into a specific position in the datasheet and results were given as positive (1) or negative (0). The negative extraction control included in every 96-well plate serves as the background reading for which all the other results are compared. Results were compiled based on the 24 hr absorbance readings. A positive result was defined as greater than 3 times the absorbance observed for the negative sample.

#### High Risk and Low Risk HPV Probes

The High Risk (HR) HPV cocktail probe consists of Digoxigenin-11-ddUTP labelled oligonucleotides specific for HPV 16, 18, 31, 33, 35, 39, 45, 51, 52, 56, 58, 59, 66 and 68. 0.5pmol of probe was required per well. 10pmol of each individual HPV-type specific Digoxigenin labelled internal oligo probe was prepared in a final volume of 1ml 1xSSC/0.5%Tween and vortexed gently. The probe cocktails were stable at -20°C for up to 1 year. Individual HR HPV probes were prepared at a concentration of 10pmol/ml and used for genotyping of all HR HPV positive samples. The Low Risk (LR) HPV cocktail probe consists of Digoxigenin-11-ddUTP labelled oligonucleotides specific for HPV 6, 11, 40, 42, 43 and 44.

The probe sequences are shown in the table below. All probes were 5’ labelled with digoxygenin.

| HPV probe | Sequence (5' to 3') |
| --- | --- |
| Probe 6 | ATCCGTAACTACATCTTCCACATACACCAA |
| Probe 11 | ATCTGTGTCTAAATCTGCTACATACACTAA |
| Probe 16 | GTCATTATGTGCTGCCATATCTACTTCAGA |
| Probe 18 | TGCTTCTACACAGTCTCCTGTACCTGGGCA |
| Probe 31 | TGTTTGTGCTGCAATTGCAAACAGTGATAC |
| Probe 33 | TTTATGCACACAAGTAACTAGTGACAGTAC |
| Probe 34 | TACACAATCCACAAGTACAAATGCACCATA |
| Probe 35 | GTCTGTGTGTTCTGCTGTGTCTTCTAGTGA |
| Probe 39 | TCTACCTCTATAGAGTCTTCCATACCTTCT |
| Probe 40 | GCTGCCACACAGTCCCCCACACCAACCCCA |
| Probe 42 | CTGCAACATCTGGTGATACATATACAGCTG |
| Probe 43 | TCTACTGACCCTACTGTGCCCAGTACATAT |
| Probe 44 | GCCACTACACAGTCCCCTCCGTCTACATAT |
| Probe 45 | ACACAAAATCCTGTGCCAAGTACATATGAC |
| Probe 51 | AGCACTGCCACTGCTGCGGTTTCCCCAACA |
| Probe 52 | TGCTGAGGTTAAAAAGGAAAGCACATATAA |
| Probe 54 | TACAGCATCCACGCAGGATAGCTTTAATAA |
| Probe 56 | GTACTGCTACAGAACAGTTAAGTAAATATG |
| Probe 58 | ATTATGCACTGAAGTAACTAAGGAAGGTAC |
| Probe 59 | TCTACTACTGCTTCTATTCCTAATGTATAC |
| Probe 66 | TATTAATGCAGCTAAAAGCACATTAACTAA |
| Probe 68 | TCTACTACTACTGAATCAGCTGTACCAAAT |

**HMBS PCR**

The HMBS primers amplify a 119bp region of the Homo sapiens hydroxymethylbilane synthase gene (GenBank accession no. M95623.1) as described in:

Real-Time PCR-Based System for Simultaneous Quantification of Human Papillomavirus Types Associated with High Risk of Cervical Cancer. Martin Moberg, Inger Gustavsson, and Ulf Gyllensten Journal of Clinical Microbiology, 2003, 41: p.3221–3228

The primer sequences (5’-3’) were:

HMBS-F GCCTGCAGTTTGAAATCAGTG

HMBS-R CGGGACGGGCTTTAGCTA

GP 5+/6+ 1 x 25 µl reaction (µl)

dNTPs 2mM 2.5

10 x PCR buffer 2.5

MgCl2 (50mM) 0.875

HMBS-F primer (5μM) 2.5

HMBS-R primer (5μM) 2.5

H2O 15.525

Taq (Invitrogen) 0.1

DNA 1

PCR cycling conditions were 94°c - 4mins, then 40 cycles of 94°c - 30s, 60°c - 30s, 72°c - 30s followed by 72°c – 4mins.
